# Functional Properties of Banana Starch (*Musa* spp.) and Its Utilization in Cosmetics

**DOI:** 10.3390/molecules26123637

**Published:** 2021-06-14

**Authors:** Norramon Thanyapanich, Ampa Jimtaisong, Saroat Rawdkuen

**Affiliations:** 1 School of Cosmetic Science, Mae Fah Luang University, 333, Moo.1, Thasud, Muang, Chiang Rai 57100, Thailand; n.nanorr@gmail.com; 2 Unit of Innovative Food Packaging & Biomaterials (IFP), Mae Fah Luang University, 333, Moo.1, Thasud, Muang, Chiang Rai 57100, Thailand; saroat@mfu.ac.th; 3 School of Agro-Industry, Mae Fah Luang University, 333, Moo.1, Thasud, Muang, Chiang Rai 57100, Thailand

**Keywords:** amylose content, banana starch, characterization, functional properties, *Musa* spp.

## Abstract

Unripe banana fruit of *Musa acuminata* (Musa AAA; Hom Khieo) and *Musa sapientum* L. (Musa ABB; Namwa) growing in Chiang Rai (Thailand) were used for extraction. The yield of the starches was 16.88% for Hom Khieo (HK) and 22.73% for Namwa (NW) based on unripe peeled banana fruit. The amylose contents of HK and NW were 24.99% and 26.23%, respectively. The morphology of starch granules was oval shape with elongated forms for large granules and round shape for small granules. The HK and NW showed B-type crystalline structure and the crystallinities were 23.54% and 26.83%, respectively. The peak temperature of gelatinization was around 77 °C and the enthalpy change (ΔH) was 3.05 and 7.76 J/g, respectively. The HK and NW banana starches showed 1.27 ± 0.12 g/g and 1.53 ± 0.12 g/g water absorption capacity, and 1.22 ± 0.11 g/g and 1.16 ± 0.12 g/g oil absorption capacity, respectively. The swelling power of the banana starches was 17.23 ± 0.94 g/g and 15.90 ± 0.15 g/g, respectively, and the percentage of solubility in water showed 26.43 ± 2.50 g/g and 20.54 ± 0.94 g/g, respectively. The banana starches showed very poor flow character. The HK and NW starches have the potential to be used in powder base preparations with no effect on the sensory texture of the product at 15% *w*/*w* maximum.

## 1. Introduction

Starch is a natural polysaccharide which is a semi-crystalline structure consisting of amylose and amylopectin. Amylose is a straight chain, whereas amylopectin has a branched chain structure. Starch can be extracted from various plant sources, especially tropical plants such as cereals, tubers, and some unripe fruits which have polysaccharide contents are in the range of 60–90% [1,2]. Bananas are edible fruit cultivars with dietary characteristics that are an important source to people [3]. The banana is a tropical fruit that belongs to the Musaceae botanical family. The *Musaceae* are monocotyledon herbaceous plants that represent hybrid-polyploid complexes of *Musa acuminata* (AA) and *Musa balbisiana* (BB), as diploids, triploids, and tetraploids [4]. Generally, bananas with a high proportion of *M. balbisiana* (B genes) tend to be more tolerate and produce more starch than bananas with high proportion of *M. acuminata* (A genes) [5]. The main component of green bananas is starch with about 70–80%, based on dry weight, but this changes dramatically during ripening from starch to sugar [6].

In Thailand, bananas are called “Kluai” and the varieties that widely cultivated in Thailand are Kluai Namwa (ABB group), Kluai Hom (AAA group), Kluai Khai (AA group) and Kluai Lep Meu Nang (AA group) [7]. Most bananas in Thailand are grown as an economic product for consumption and exportation, with an estimated amount of 27,452 tons of bananas exported in 2011–2019. Damaged or too-small bananas will be culled and become waste. There is an attempt to bring the culled bananas to be used as animal feeds, but these products have low value. The production of starch from the banana culls can improve banana economics and eliminate environmental problems [8]. Natural starch plays an important role in various industries such as the food, pharmaceutical, and cosmetic industries. It can be used both as powdered starch and gelatinized starch. Generally, starch provides smoothness when rubbed on the skin and can improve the smoothness of products [9,10]. Starch also act as a thickening agent, gelling agent, and stabilizing agent. The thickening agent from starch is effective in stabilizing emulsions to avoid phase separation [11,12,13]. Starch is also used in powder formulation, according to Rincón et al. [14], guapo starch has been used in the preparation of face powders as a substitute for talcum, and a satisfaction rating was assessed against a base formulation. Sago starch has also been used in body powder formulations. Satisfaction assessments of perfumed and cooling body powders containing sago starch were similar to the commercial formulation [15]. Thus, it is interesting to find an alternative source of natural starch as a substitute for talcum and use as an ingredient in cosmetic preparation.

Recently, we have preliminarily reported the basic properties such as morphology, amylose content, crystalline structure and the thermal properties of *M. acuminata* (AAA group) [16]. In this study, the banana starch from Namwa (NW), a member of *sapientum* subgroup in the ABB group, was investigated alongside, and compared to, the HK starch in terms of yield and properties. Additionally, the utilization of starch as an ingredient in cosmetic formulation was also described.

## 2. Results and Discussion

### 2.1. Extraction of Banana Starch

The percentage yield of HK and NW that was obtained from extraction with 1% sodium sulfite was 16.88 and 22.73%, respectively, calculated based on fresh peeled banana. It can be seen that NW (Musa ABB) had higher yield than HK (Musa AAA), as it has been reported that bananas with high proportion of B genes tend to produce more starch than that with high proportion of A genes [4]. However, the yields of HK and NW starches were in the normal range compared with those found in previous studies. According to Bello-Perez et al. [17], the yields of *M. paradisiaca* (AAB) and *M. acuminata* (AAA) were 43.8% and 11.8%, respectively. Chávez-Salazar et al. [18] reported that the yield of *M. paradisiaca* (AAB), and *M. acuminata* (AAA) were 32.33% and 18.73%, respectively. The variety and texture of fruit affect the yield of starch, lower firmness results in lower yield than higher firmness, which is related to the stage of ripeness [8,17].

**Figure 1:**
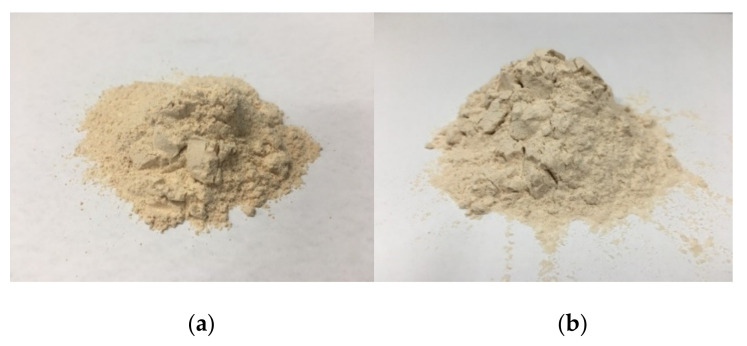
Appearance of banana starch (**a**) *M. acuminata* (AAA; HK) (**b**) *M. sapientum* L. (ABB; NW).

The starches showed light yellow color (Figure 1) with fine texture and light characteristic banana odor. The color values that obtained from the colorimeter in term of L*, a*, and b* are 82.67, 2.83, and 10.92, and 89.57, 1.61, and 9.03 for HK and NW, respectively. The color values of HK banana starch obtained were similar to those reported by Falade & Oyeyinka [19] as L*, a*, and b* of Agbagba (Musa AAB), Cooking bananas (Musa ABB), and Obino I’Ewai (Musa AAB) were in range of 81.30–82.03, 1.82–2.18, and 11.11–12.19, respectively. Whereas, NW was slightly lighter, less green, and less yellow.

### 2.2. Amylose Content

Starch is a polysaccharide, consisting of amylose and amylopectin. In general, common starch constitutes of 20–30% amylose and 70–80% amylopectin [20,21]. Amylose contents of starch granules extracted from HK and NW were 24.99 ± 1.03% and 26.23 ± 0.27%, respectively. The amylose contents of HK and NW were similar to those found in a previous study by Barros Mesquita et al. [22], in which amylose content of Musa AAA (25.31–26.54%) was slightly lower than Musa AAB (26.42–29.01%). The results reported in the study of Utrilla-Coello et al. [23] showed that the amylose content of Musa AAA (19.32–21.99%) was lower than Musa AAB (26.35%) and Musa ABB (25.38%). The amylose content plays an important role in influencing diverse physicochemical and functional properties of starches. The differences of amylose content depend on the variety, growth condition and the state of maturity of the plant [18,21,22].

### 2.3. Moisture Content

The percentage of moisture content of HK and NW were 13.10 ± 0.10% and 13.40 ± 0.40%, respectively. The moisture content was similoar to that found by Barros Mesquita et al. [22] which reported the moisture content of Musa AAA and Musa AAB in a range of 8.60–14.00%. The moisture content affected the properties of starch as higher moisture content has the ability to bind with water but low ability to flow [24,25].

### 2.4. Morphology

**Figure 2:**
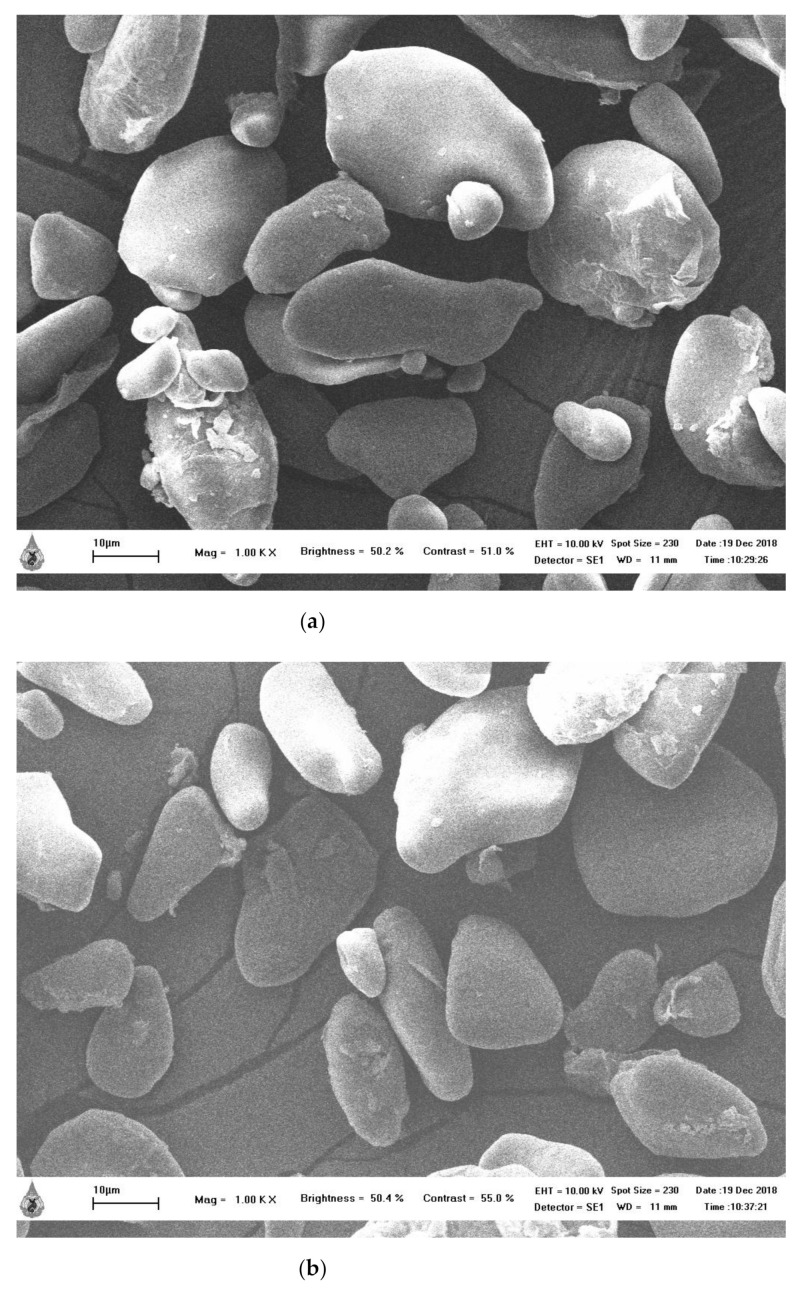
The morphology of banana starch at 1000× (**a**) *M. acuminata* (AAA; HK) (**b**) *M. sapientum* L. (ABB; NW).

The HK and NW starch granules exhibited various sizes and shapes. The large size of both HK and NW starches was an oval shape with elongated form. The average size of HK was around 39 µm, which was close to that of NW (37 µm), while the smaller size of HK and NW was round shape with a size around 10 µm. The HK and NW granules had a higher proportion of large sizes than smaller sizes. The appearance of granules from the SEM showed that their surfaces were smooth and dense (Figure 2).

The banana starch granules were similar to the previous studies of various cultivars which were irregular elongated oval shape with dense and smooth surfaces [23,26,27,28,29]. However, both the size and shape of the banana starch granules depends on the banana cultivar. Some cultivars were rod, or round with clefts shaped [30]. The size of the HK and NW starch granules in this study is in the range of that found by Bello-Pérez et al. [26] ranging between 35–55 µm, while the small size was around 7–8 µm.

The morphology of starch depends on the botanical source that affects the shapes and size of starch granules. Starch granules of corn are angular-shaped with 1–7 µm for small granules and 15–20 µm for large granules. Rice starch granules are pentagonal with angular-shapes and the granules sizes range from 3 to 5 µm. Whereas potato starch granules are oval and irregular shapes with 1–20 µm for the granule size. The SEM images of potato starch granules are smoother than those of corn and rice starch granules [31]. In this work, the banana starch granules showed smoother surfaces than corn and rice starch, similar to the potato starch granules that give the smooth feeling. HK and NW starch granules were larger than corn, rice, and potato starch. However, the variety of granule size and shape depends on the starch source that affected the physicochemical and functional properties of the starch [30,31].

### 2.5. FT-IR Spectroscopy

**Figure 3:**
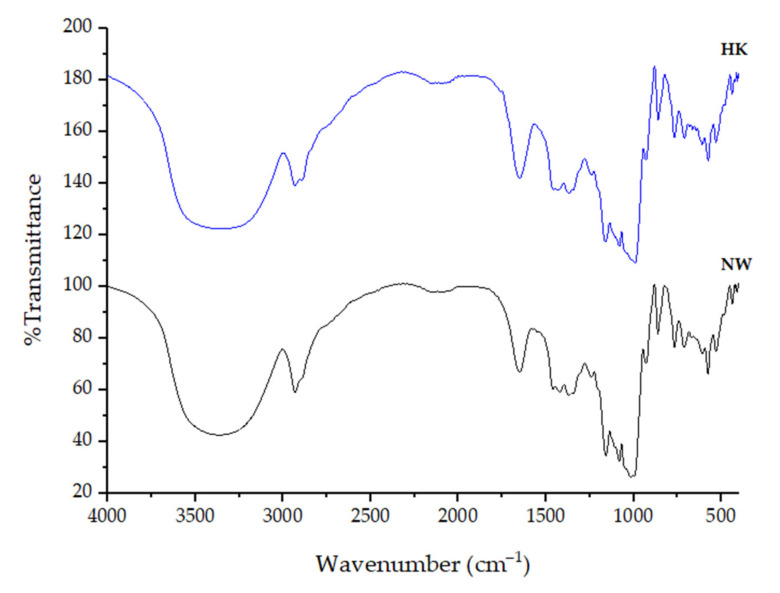
FT-IR of *M. acuminata* (AAA; HK) and *M. sapientum* L. (ABB; NW) starches.

The FT-IR spectra of HK and NW starch samples are shown in Figure 3. The spectra showed peaks at around 3300 cm^−1^ (O-H stretching); 2900 cm^−1^ (C-H stretching); 1650 cm^−1^ (C-O associated with OH group); 1424 cm^−1^ (CH_2_ symmetric deformation); 1370 cm^−1^ (C-H symmetric bending); 1160 cm^−1^ (C-O-C asymmetric stretching); 1082 cm^−1^ and 1000 cm^−1^ (C-O stretching); and 930 cm^−1^, 860 cm^−1^, and 767 cm^−1^ (C-O-C ring vibration of carbohydrate). The FT-IR spectra obtained from this study were similar to those from a previous report [20].

### 2.6. X-Ray Diffractometer (XRD)

Starch is a semi-crystalline material affected by amylose content and amylopectin chain length that consists of amorphous and crystalline regions. The crystalline region is parallel of glucan chains (amylopectin) that are closely packed and ordered, whereas the amorphous region is the linear region (amylose). The amylose content directly affects the crystallinity degree of the starch, such that when there is a lack of amylose content, the crystallinity degree increases, whereas the longer chain amylopectin forms have a more stable crystalline structure [32,33]. Generally, the starch crystalline structure was divided into three types, which are A-type, B-type, and C-type that depended on the variety of starch source. The crystalline structure was determined by using the XRD technique. A-type starch showed strong peaks at a 2*θ* of about 15°, and 23° and small peaks at about 17° and 18°. However, the strong peaks of B-type presented at about 17° and small peaks at about 15°, 20°, 22°, and 24° with a characteristic peak at about 5°. The peaks of C-type starch were a mixture of A-type and B-type at 2*θs* of about 17° and 23° and small peaks at 5° and 15° [34,35,36].

**Figure 4:**
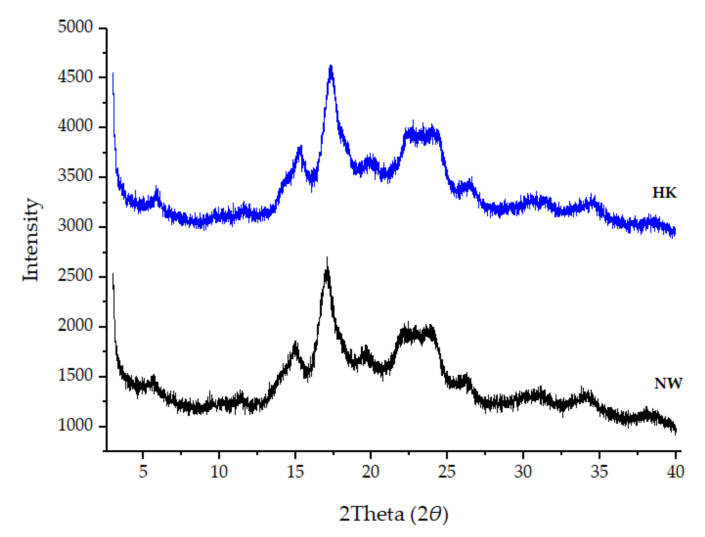
X-ray diffraction pattern of HK and NW starch granules.

The X-ray diffraction pattern of HK was similar to that of NW starches (Figure 4). The HK showed a pattern with major peaks at 2*θs* of about 15.12°, 17.07° and a doublet at 22.13° and 23.64°, with weak diffraction peaks at 5.31° and 19.83°, with 23.54% crystallinity. The NW was identified by major diffraction peaks at 2*θs* of 15.30°, 17.22°, and a doublet at 22.70° and 23.90° with weak diffraction peaks at 5.76° and 19.81°, with 26.84% crystallinity. From the X-ray pattern of the HK and NW starch granules, they were determined to be B-type starches.

The major difference between A- and B-types of starch granules is the arrangement of double helices. A-type starches form dense packing with 4 water molecules, whereas B-type starch is more open causing more water molecules (36 water molecules) to be located in the center of a hexagonal packing of helices. For this reason, it is indicated that the A-type is more stable and requires a higher temperature than B-type starch for gelatinization [11,34,37].

### 2.7. Thermal Properties

The differential scanning calorimetry (DSC) and gelation methods were used to determine the thermal properties of banana starches. The thermal properties from DSC were measured in terms of transition temperature; onset (To), the starting point of starch gelatinization; peak (Tp), the point where a complete loss of crystalline occurs; and end set (Te), the final temperature required to complete the gelatinization. The enthalpy (ΔH) is consistent with the changes of energy during melting of the crystallites and measures the crystallinity or damage in the starch structure before gelatinization occurs [38,39,40].

The HK banana starch has an onset gelatinization temperature (To) of 74.52 °C, a peak gelatinization temperature (Tp) of 77.97 °C, and an endpoint gelatinization temperature (Te) of 80.37 °C, with gelatinization enthalpy (ΔH) of 3.05 J/g. The NW banana starch has an onset gelatinization temperature (To) of 73.64 °C, a peak gelatinization temperature (Tp) of 76.98 °C, and an endpoint gelatinization temperature (Te) of 80.69 °C, with gelatinization enthalpy (ΔH) of 7.76 J/g (Table 1). The results were in agreement with a previous study that found the gelatinization temperatures of banana starches are in a range of 70–79 °C. The enthalpy (ΔH) of NW was higher than that of HK, indicating that NW had a higher proportion of crystalline structure than HK starch, which is related to the results of crystallinity [1,18,23,26,41,42,43].

**Figure 5:**
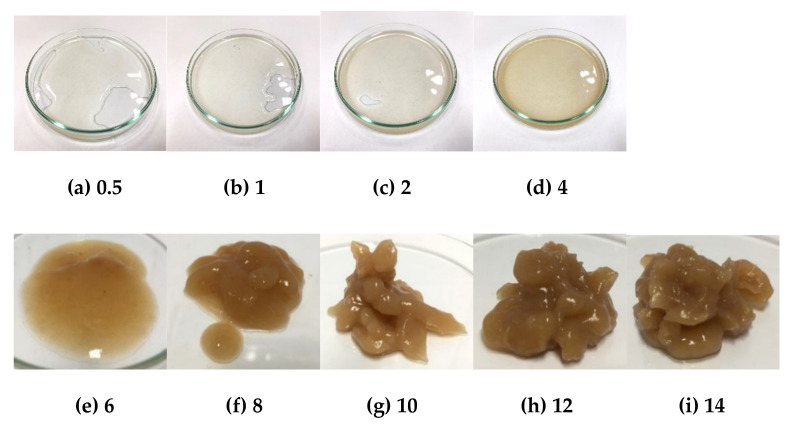
Gelation of HK banana starch at 80 °C at different concentrations where (**a**) 0.5% *w*/*v*, (**b**) 1% *w*/*v*, (**c**) 2% *w*/*v*, (**d**) 4% *w*/*v*, (**e**) 6% *w*/*v*, (**f**) 8% *w*/*v*, (**g**) 10% *w*/*v*, (**h**) 12% *w*/*v*, and (**i**) 14% *w*/*v*.

**Figure 6:**
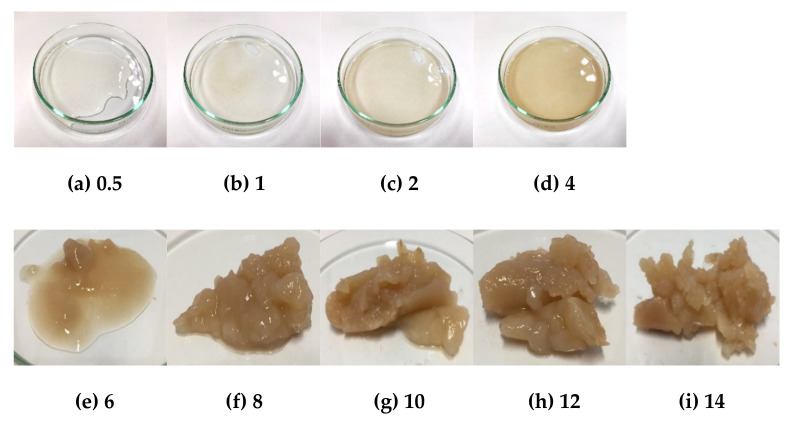
Gelation of NW banana starch at 80 °C at different concentrations where (**a**) 0.5% *w*/*v*, (**b**) 1% *w*/*v*, (**c**) 2% *w*/*v*, (**d**) 4% *w*/*v*, (**e**) 6% *w*/*v*, (**f**) 8% *w*/*v*, (**g**) 10% *w*/*v*, (**h**) 12% *w*/*v*, and (**i**) 14% *w*/*v*.

According to Mar et al. [44] the high degree of crystallinity is the cause of the high transition temperature values of starches, which provides structural stability and makes the starch granules more resistant to gelatinization. The higher percentage crystallinity of amylopectin requires the higher gelatinization enthalpy (ΔH) [45,46]. For the gelation method, the starch concentration was varied (0.5, 1, 2, 4, 6, 8, 10, 12, and 14% *w*/*v*) at different temperatures (55, 65, 75, and 80 °C) and it was found that all samples at temperatures lower than 80 °C with low concentrations (0.5–6% *w*/*v*) did not show gel formation and high concentrations (8–14% *w*/*v*), showed some of gel forming. At 80 °C, the gelation of HK and NW starches are shown in Figure 5 and Figure 6, they presented that the concentrations of 0.5 to 6% *w*/*v* showed some gel forming. At 8% *w*/*v* the starch presented the gel formation stage. The banana starch showed complete gel formation at 10 to 14% *w*/*v*. The results indicated that the gelatinization temperatures of HK and NW obtained from the DSC method were related to those found by the gelation method.

### 2.8. Water and Oil Absorption Capacities

The water absorption capacities (WAC) of HK and NW were 1.27 ± 0.12 g/g and 1.53 ± 0.12 g/g whereas the oil absorption capacities (OAC) of HK and NW were 1.22 ± 0.11 g/g and 1.16 ± 0.12 g/g, respectively. The WAC represents the ability of a substance to associate with water under a limited water condition. Agnes et al. [47] reported that water absorption capability is useful to indicate that starch granules can be associated with aqueous formulations.

### 2.9. Swelling Power and Solubility

**Figure 7:**
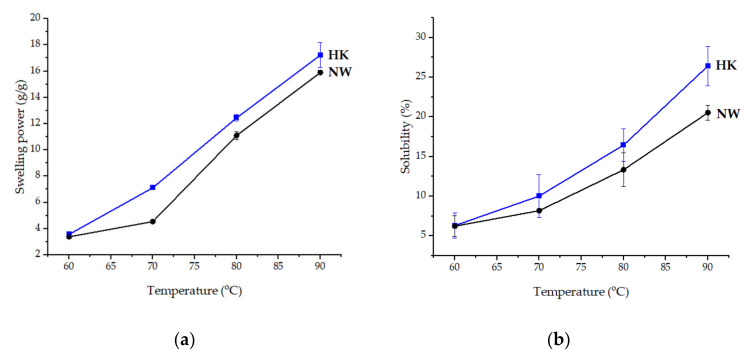
(**a**) swelling power of HK and NW banana starches (**b**) solubility of HK and NW banana starches.

The swelling power and solubility of HK and NW were directly related to increases in temperature. Both HK and NW starches showed a two-stage process. The initial stage was in range of 60–70 °C where the starches exhibited low swelling, then a significant increase showed above 70 °C, which is in agreement with previous studies [48,49,50]. At temperatures above 70 °C, both HK and NW swelled rapidly until 90 °C (17.23 ± 0.94 g/g and 15.90 ± 0.15 g/g) that showed the highest swelling power (Figure 7a). During the initial stage, the starches exhibited slight swelling due to hydrogen bonds in the starch granules forming a complex of lipids and proteins that restricted swelling. At above 70 °C, the hydrogen bonds were disrupted and allowed water penetrated into the starch granules that increased swelling [51]. 

Amylose content maybe one of the factors affecting the swelling power of HK and NW starches due to the lower amylose content of HK causing the higher swelling power than the NW starch. HK and NW banana starch solubility also increased with temperature, reaching 26.43 ± 2.50% and 20.54 ± 0.94% at 90 °C, respectively (Figure 7b). The HK starch has slightly higher solubility than NW due to the lower amylose content, which is in agreement with Rodríguez-Torres et al. [52].

The starch granule is a water-insoluble compound that can be hydrated at high temperatures. The disorder of starch crystallinity from heating can hydrate and swell the starch granules [39]. The swelling power is dependent on the granule structure that is obtained from different starch source. The broken of H-bonds from heating allowed the swelling of the granules. The swelling power depends on the water holdeing capacity via amylose content, chains of amylopectin, and H-bonds [18,53]. The high amylopectin content causes high swelling power and high viscosity at low temperature [54]. The pores on the starch surface allow water to penetrate and interrupt the amylose in amorphous regions and improves the swelling power of starch granules [55]. The size of the starch granules also effects the swelling power as the smaller size causes higher swelling power. The small size starch granules have a high amylopectin content which is highly crystalline and allows more swelling power [18,38]. Amylose content affected the swelling power and solubility in an inverse relationship. The high swelling power was due to the low amylose content, which is in accordance with the results of Mtunguja et al. [56], who reported that the higher amylose content causes low swelling power of cassava starches and agreed with Sánchez et al. [57] that the waxy cassava starches had the highest swelling power.

### 2.10. Flow Properties

The flow character of HK and NW banana starches was obtained from examination and compared with the compressibility index (CI) and Hausner ratio (HR) chart (Table 2). The HK and NW banana starches showed very poor flow properties with 31.70 ± 2.13 and 34.84 ± 1.59 compressibility indeces, and 1.47 ± 0.05 and 1.54 ± 0.04 Hausner ratios, respectively. Generally, the moisture content and water absorption capacity affect the flowability of starches and are always inversely correlated. The higher moisture content and water absorption capacity causes more water absorption which results in increased cohesion and decreased flowability [24,58,59,60,61]. The particle size of starches also influences the flowability in that fine or small sized granules flowed less freely than large sized granules and the presence of a high proportion of fine granules reduces the flowability. The very poor flowability of the NW starch showed higher values in the compressibility ratio and Hausner ratio that are related to its higher water absorption capacity than the HK starch.

### 2.11. Cosmetic Formulation

Currently, the trend of using natural materials in the cosmetic industry is on the rise. Consumers demand natural ingredients to replace synthetic ingredients because synthetic ingredients can have possible negative effects on health. For this reason, to satisfy the consumer, industry needs to develop cosmetics based on natural ingredients [62,63]. According to previous research, it has been found that asbestos can be found in powders containing talcum powder, which negatively affects the respiratory system and contributes to ovarian cancer. At present, powder formulations have been developed to have a lower amount of talcum by replacing it with natural starch [64,65,66]. The powder base preparation was chosen due to the smooth surface of starch granules and the smoothness when applied on skin, a preliminary study was conducted on body powder and compact powder.

**Figure 8:**
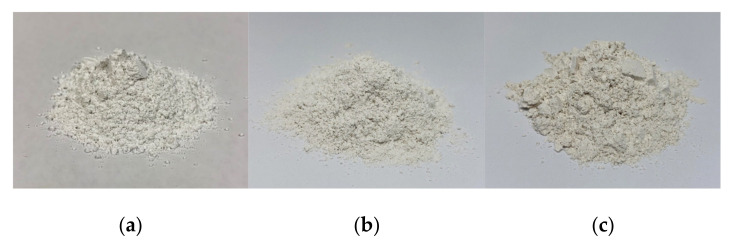
Appearance of body powder (**a**) base body powder (**b**) containing *M. acuminata* (AAA) (**c**) containing *M. sapientum* L. (ABB).

**Figure 9:**
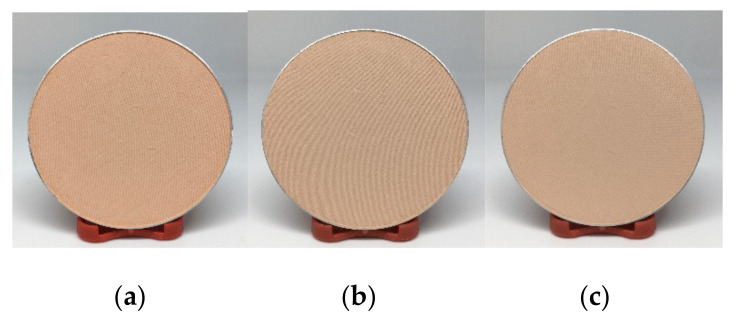
Appearance of compact powder (**a**) base compact powder (**b**) containing *M. acuminata* (AAA) (**c**) containing *M. sapientum* L. (ABB).

Body powder and compact powder containing HK or NW in a range of 5 to 20% were prepared. It was found that the products with a starch component in the range of 5 to 15% provided a smooth and soft touch similar to that of the base formulations whereas at higher than 15%, it has a less smooth and soft touch. The 15% HK and NW body powder showed a light-yellow color (Figure 8) with light banana odor. For 15% HK and NW compact powder, they showed a beige color (Figure 9) due to other colorants in the formula, with a light banana odor. It was found that when HK or NW starch was added into the formulation, it provided smoothness and could be used as a substitute for talcum powder. A sensory test in volunteers should be studied further. In addition, the flow properties of a body powder containing 15% HK and NW were similar to the base formulation (Table 3). From these results, the HK and NW starch has the potential to be used as a substitute for talcum in powder base preparation. However, the HK and NW starches needs to be modified to reduce their water absorption, which may improve the smoothness of product when use in higher quantities in powder formulation.

## 3. Materials and Methods

### 3.1. Extraction of Banana Starch

The extraction method for the banana starches was a slight modification of the method used by Bello-Pérez et al. [17]. Two varieties of unripe banana which are Hom Khieo (*M. acuminata*; AAA) and Namwa (*M. sapientum*; ABB) banana cultivars were used. The unripe bananas were peeled and cut into small cube pieces then placed into a blender and 1% *w*/*v* of sodium sulfite solution was added to immerse the banana in a ratio of 1:2 *w*/*v*. The samples were blended in the blender at maximum speed for 10 min then filtered with 200 mesh sieving and white cloth to remove the pulp. After being sieved, the samples were centrifuged at 8500 rpm for 5 min to precipitate the starch. The precipitated starches were dried at 40 ℃ in a hot air oven overnight. The dried banana starches were ground by using mortar and pestle then sieved with a 125 µm mesh. The percentage of yield of banana starch was calculated as

(1) $$\mathrm{The}\;\mathrm{percentage}\;\mathrm{of}\;\mathrm{yield}=\frac{\mathrm{Weight}\;\mathrm{of}\;\mathrm{dry}\;\mathrm{starch}}{\mathrm{Initial}\;\mathrm{weight}\;\mathrm{of}\;\mathrm{fresh}\;\mathrm{peeled}\;\mathrm{unripe}\;\mathrm{banana}\;\mathrm{fruit}}\times \;100$$

The color of Hom Khieo (HK) and Namwa (NW) starch was measured by colorimeter (Hunter Lab, Reston, VA, USA). The color was determined by the international color scale (International Commission on Illumination) which is in terms of CIELAB [67].

### 3.2. Amylose Content

Total amylose content was determined by using the colorimetric method described by Kaufman et al. [68]. The starch samples or standards (5 mg) were weighed into a microcentrifuge tube. 1 mL of 90% DMSO in distilled water was added into the samples and heated for 60 min at 95 ℃, then shaken every 10 min with a vortex. The samples were cooled down for 5 min, then 100 µL of each sample was placed into a 96-well plate. Both amylose and amylopectin were used to prepare a standard curve by varying the ratio (Table 4), and placed into the 96-well plate. Then, 100 µL of 90% DMSO in distilled water with 3.04 g/L of iodine was added into each well, and the plate was shaken for 2 min. The 2 µL of each well was transferred to the empty plate then 180 µL of distilled water was added into each well and shaken for 2 min before being analyzed with absorbance at 620 nm. The 100 µL of DMSO with 3.04 g/L and 100 µL of 90% DMSO was used for the control blank. The amylose content was calculated as the following equation:

(2) $$\mathrm{Amylose}\;\mathrm{content}=\frac{\mathrm{Absorbance}\;\mathrm{at}\;620\;\mathrm{nm}\;-\;y-\mathrm{intercept}\;\mathrm{of}\;\mathrm{regression}}{\mathrm{Slope}\;\mathrm{of}\;\mathrm{regression}}$$

### 3.3. Moisture Content

The moisture content of the starch samples was determined by using a moisture analyzer (Ohaus, Parsippany, NJ, USA). One gram of each starch sample was placed into the moisture analyzer in condition of 100 °C for 1 min then the percentage of moisture content was recorded.

### 3.4. Morphology

The morphologies of HK and NW banana starches were determined by using a scanning electron microscopy (SEM) with SEI detector. The starch samples were placed on double-side adhesive carbon tape and coated with a gold layer. The starch sample granules were measured at 1000× magnification. The sizes of HK and NW starch granules were determined by using TESCAN Essence software (Zeiss, Jena, Germany).

### 3.5. FT-IR Spectroscopy

The FT-IR spectra of sample starches were recorded by spectrophotometer (PerkinElmer, Waltham, MA, USA) using the potassium bromide (KBr) pellets method. The finely ground samples were mixed with dry KBr then pressed under high pressure to form 7 mm pellets then the sample was measured in the range of 4000–400 cm^−1^ with a resolution of 4 cm^−1^ and 16 scans, at room temperature [69].

### 3.6. X-Ray Diffractometer (XRD)

The crystallinity was measured by using an X-ray diffractometer (Malvern Panalytical, Malvern, UK) with a slightly modified version of the method used by Cheetham and Tao [34]. The HK and NW samples were packed tightly in aluminum cells then the intensities of samples were measured using the X-ray diffractometer with Cu Kα radiation (λ = 1.542), in the 3–40° 2*θ* range with a 0.01° step size and a measuring time of 10.0 s per point. The measurements were made at ambient temperature. The percentage crystallinity was calculated from the following equation [70].

(3) $$\mathrm{Crystallinity}\;(\%)=\frac{\mathrm{Ac}}{\mathrm{Ac}+\mathrm{Aa}}\times \;100$$

where Ac = crystalline area and Aa = amorphous area.

### 3.7. Differential Scanning Calorimetry (DSC)

The thermal properties of the samples were analyzed by using differential scanning calorimetry with slightly modified version of the method used by Carmona-Garcia et al. [5]. Seven mg of each starch sample was weighed into an aluminum pan (40 µL) then 20 µL of distilled water was added and sealed tightly. The suspensions were allowed to stand at room temperature for 1 h before analysis. The samples were subjected to the DSC at a range of temperatures from 40 to 90 °C and a heating rate of 10 °C /min under N2 atmosphere. The empty aluminum pan was used as a reference.

### 3.8. Gelation

Gelation of starch samples was determined by a slight modification of the method used by Lawal and Adebowals [71]. Starch samples were prepared at 0.5, 1, 2, 4, 6, 8, 10, 12, and 14% *w*/*v* in test tubes with distilled water. The suspensions were mixed with a vortex until homogenous. The test tubes were heated in a water bath at 55, 65, 75, and 80 °C for 30 min and cooled down with cold running tap water then cooled at 4 °C for 2 h. The least gelation concentration was determined by the concentration when the test tube was inverted, and the sample did not slip or fall-down.

### 3.9. Water and Oil Absorption Capacities

A slightly modified version of the method used by Agnes et al. [47] was used to determine the water and oil absorption capacity. Ten mL of water or soybean oil was added to 1 g of each starch sample. The suspension was allowed to stand at room temperature for 30 min then centrifuged at 3500 rpm for 30 min. The supernatant was measured using a 10 mL cylinder. The difference between the initial water or oil used and the volume of the supernatant obtained after centrifuging was used to calculate the water or oil absorbed by the starch sample. The result was expressed as g/mL of water or oil absorbed.

### 3.10. Swelling Power and Solubility

Swelling and solubility of the starch samples were determined by a slight modification of the method used by Carmona-Garcia et al. [5]. 1% *w*/*v* of starch solution was prepared with distilled water in centrifuge tubes and water loss was prevented by covering the tubes with plastic caps. The tubes were then heated at 60, 70, 80, and 90 °C for 30 min with shaking every 5 min. After heating, the samples were centrifuged at 8000 rpm for 5 min. The supernatant was decanted, then both of supernatant and precipitate were dried at 100 °C overnight. The swelling power and solubility of the samples were determined by the following equation.

(4) $$\mathrm{Swelling}\;\mathrm{power}=\frac{\mathrm{Weight}\;\mathrm{of}\;\mathrm{swollen}\;\mathrm{granules}}{\mathrm{Weight}\;\mathrm{of}\;\mathrm{precipitate after}\;\mathrm{dry}}$$

(5) $$\mathrm{Solubility}=\left(\frac{\mathrm{Weight}\;\mathrm{of}\;\mathrm{solids}\;\mathrm{in}\;\mathrm{supernatant}}{\mathrm{Weight}\;\mathrm{of}\;\mathrm{dry}\;\mathrm{both}\;\mathrm{of}\;\mathrm{precipitate}\;\mathrm{and}\;\mathrm{supernatant}}\right)\times \;100$$

### 3.11. Flowability

#### 3.11.1. Bulk and Tapped Densities

Measurement of the bulk, and tapped densities followed the method of Olayemi et al. [72]. Fifty grams (Wp) of the starch samples were gently poured through a glass funnel into a 100 mL cylinder. The volume occupied by the sample was taken as Vp. The powders were tapped on a wooden surface at height of 7 inches until no further change in volume was observed (VpT).

(6) $$\mathrm{Bd}=\frac{\mathrm{Vp}}{\mathrm{Wp}}$$

(7) $$\mathrm{Td}=\frac{\mathrm{VpT}}{\mathrm{Wp}}$$

where Bd = Bulk density and Td = Tapped density.

#### 3.11.2. Compressibility Index and Hausner Ratio

The bulk and tapped densities were used to calculate the compressibility index and the Hausner ratio to provide a measure of the flow properties [72,73].

(8) $$\mathrm{Hausnerratio}\;(\mathrm{HR})=\frac{\mathrm{Bd}}{\mathrm{Td}}$$

(9) $$\mathrm{Compressibility}\;\mathrm{index}\;(\mathrm{CI})=\frac{\left(\mathrm{Bd}-\mathrm{Td}\right)}{\mathrm{Bd}}\times \;100$$

### 3.12. Preparation of Cosmetic Formulations

Two powder formulations (body powder and compact powder) were prepared by varying the concentration of HK and NW starches to find the highest concentration suitable for the formulations. Body powder was composed of 5 to 20% HK or NW banana starch, talcum, magnesium carbonate, kaolin, zinc stearate, and zinc oxide. 1% of phenoxyethanol as a preservative was added to formula. Compact powder was composed of 5 to 20% HK or NW, talcum, kaolin, zinc stearate, zinc oxide, titanium dioxide coated mica, magnesium stearate, and magnesium carbonate. Titanium dioxide, red iron oxide, yellow iron oxide, and black iron oxide as a colorant, 0.5% of capric/caprylic triglyceride, 1% of mineral oil, and 1% of isopropyl myristate as binding agents, and 1% of phenoxyethanol as a preservative were added to formula.

## 4. Conclusions

The banana starch of Hom Khieo (HK) and Namwa (NW) cultivars were successfully extracted with 1% sodium sulfite with a high yield based on unripe peeled banana fruit. The starches showed light-yellow color with a fine powder. The morphology of both starches was oval shape with elongated forms for large granules and round shapes for small granules. The amylose content of HK and NW were 24.99% and 26.23%, respectively. Each starch showed the B-type crystalline structure and the gelatinization temperature was around 77 °C. The banana starches showed very poor flow character and provide smoothness when applied on skin. The HK and NW banana starch were used in powder formulations to provide a smooth feel and good spreading properties. The starch has the potential to be used as a substitute for talcum in powder base preparation with no effect on sensorial properties at 15% *w*/*w* maximum.

## Figures and Tables

**Table 1:** Thermal properties of HK and NW banana starch obtained using DSC technique.

Starch	To (°C)	Tp (°C)	Te (°C)	ΔH (J/g)
HK	74.52 ± 0.17	77.97 ± 0.21	80.37 ± 0.50	3.05 ± 0.83
NW	73.64 ± 0.32	76.98 ± 0.21	80.69 ± 0.23	7.76 ± 0.74

**Table 2:** The relationship between Compressibility index, Hausner ratio, and flowability.

Flow Character	HR	CI (%)
Excellent/very free flow	1.00–1.11	10
Good/free flow	1.12–1.18	11–15
Fair	1.19–1.25	16–20
Passable	1.26–1.34	21–25
Poor/cohesive	1.35–1.45	26–31
Very poor/very cohesive	1.46–1.59	32–37
Very very poor/approx. non-flow	>1.60	>38

**Table 3:** The flow properties of the body powder containing 15% starch.

	Base Body Powder	HK Body Powder	NW Body Powder
Density	0.88 ± 0.02	0.84 ± 0.03	0.86 ± 0.01
Hausner ratio	1.84 ± 0.05	1.78 ± 0.06	1.86 ± 0.03
Compressibility index	45.85 ± 1.50	43.67 ± 1.95	45.62 ± 0.59

**Table 4:** Standard curve preparation.

Amylose Content (%)	Amount of 5 mg/mL Amylose Solution (µL)	Amount of 5 mg/mL Amylopectin Solution (µL)
0	0	100
5	5	95
10	10	90
15	15	85
20	20	80
25	25	75
30	30	70
50	50	50
75	75	25
100	100	0

## Data Availability

All the data are available in the manuscript.

## Sample Availability

Samples of the compounds HK and NW starch are available from the authors.
